# Postsynaptic Odorant Concentration Dependent Inhibition Controls Temporal Properties of Spike Responses of Projection Neurons in the Moth Antennal Lobe

**DOI:** 10.1371/journal.pone.0089132

**Published:** 2014-02-19

**Authors:** Terufumi Fujiwara, Tomoki Kazawa, Stephan Shuichi Haupt, Ryohei Kanzaki

**Affiliations:** 1 Graduate School of Information Science and Technology, The University of Tokyo, Tokyo, Japan; 2 Research Center for Advanced Science and Technology, The University of Tokyo, Tokyo, Japan; INRA-UPMC, France

## Abstract

Although odorant concentration-response characteristics of olfactory neurons have been widely investigated in a variety of animal species, the effect of odorant concentration on neural processing at circuit level is still poorly understood. Using calcium imaging in the silkmoth (*Bombyx mori*) pheromone processing circuit of the antennal lobe (AL), we studied the effect of odorant concentration on second-order projection neuron (PN) responses. While PN calcium responses of dendrites showed monotonic increases with odorant concentration, calcium responses of somata showed decreased responses at higher odorant concentrations due to postsynaptic inhibition. Simultaneous calcium imaging and electrophysiology revealed that calcium responses of PN somata but not dendrites reflect spiking activity. Inhibition shortened spike response duration rather than decreasing peak instantaneous spike frequency (ISF). Local interneurons (LNs) that were specifically activated at high odorant concentrations at which PN responses were suppressed are the putative source of inhibition. Our results imply the existence of an intraglomerular mechanism that preserves time resolution in olfactory processing over a wide odorant concentration range.

## Introduction

How sensory information on physical or chemical quantities is processed in neuronal circuits is a fundamental question in understanding sensory systems. While odorant concentration-response characteristics of olfactory neurons have been well investigated in a variety of animal species [1]–[7], the effect of odorant concentration on changes of the representation of individual odorants from one processing level to the next in olfactory circuits is not well understood. It has recently been demonstrated in the *Drosophila* antennal lobe, the primary olfactory center of the insect brain, that spike firing rates of second-order projection neurons (PNs) are suppressed in a concentration-dependent manner, which enhances the difference of PN response amplitudes among glomeruli over a wide concentration range, promoting concentration-invariant odorant discrimination [8], [9]. As far as odorant discrimination is concerned, presynaptic lateral inhibition plays an important role in the communication among glomeruli [8], [10], [11].

Still, odorant concentration is a key parameter for animals to localize odorant sources [12], [13]. In this behavioral task, the information concerning specific odorants of interest may mainly be processed by an intraglomerular circuit, in particular for odorants encoded by a labeled-line scheme such as pheromones. Pheromone-processing circuits of male moths are simple, convenient systems to test the significance of intraglomerular mechanisms due to the well-defined component-specific processing of pheromone information [14]. Each female sex pheromone component activates one type of ORN innervating a corresponding glomerulus in the macroglomerular complex (MGC) of the AL [15], [16]. The information is then processed by PNs and local interneurons (LNs) in the AL. Although concentration-response characteristics of MGC neurons have been investigated previously [6], [17]–[20], mechanisms adjusting processing at circuit level depending on stimulus concentration remain to be elucidated.

Using male silkmoths, we investigated the neuronal response properties in the AL for a series of concentrations of the major female sex pheromone component ((*E*, *Z*)-10,12-hexadecadienol, bombykol), which is represented in the toroid glomerulus [15], [21], using calcium imaging and loose-patch voltage recording to unravel the involvement of the concentration parameter in the processing of an individual odorant in an intraglomerular circuit of the AL. We found that PN spike response duration rather than peak instantaneous spike frequency (ISF) was reduced by postsynaptic inhibition for high odorant concentration stimuli. In addition, LNs, the putative origin of inhibition, showed corresponding increased responses at high odorant concentrations. This mechanism allows PNs to encode temporal properties of the stimulus while also encoding concentration information over a wide range of concentrations in their peak firing rate.

## Materials and Methods

### Animals

Male silkmoth larvae (*Bombyx mori* L.) were reared on artificial diet (Silk Mate 1–3S; Nosan Corporation Bio Department) at 26°C and 60% relative humidity under a 16∶8 h (light/dark) light cycle or obtained as pupae (Nikko Marketing). Adult moths were used 1–4 d post eclosion.

### Animal Preparation

The abdomen, legs, and dorsal side of the thorax were removed and the moths were mounted in a holder. The brain was exposed and superfused with saline solution (140 mM NaCl, 5 mM KCl, 7 mM CaCl_2_, 1 mM MgCl_2_, 4 mM NaHCO_3_, 5 mM trehalose, 5 mM N-tris [hydroxymethyl] methyl-2-aminoethanesulfonic acid, and 100 mM sucrose, pH7.0). Intracranial muscles, parts of the compound eyes, mouth parts and tracheae around the AL were removed and the AL was surgically desheathed.

### Imaging

A calcium indicator was loaded into PNs as described previously [22]. A micropipette (2 µm inner diameter), filled with Calcium Green dextran (3000MW, C-6765; Molecular Probes, 5% in water) was inserted into the outer region of the toroid innervated by the dendrites of bombykol responsive PNs. Using iontophoresis with cathodal current (20 µA amplitude, 60 pulses with 25 ms pulse duration at 500 ms interval through the pipette using an Ag/AgCl wire in the saline solution covering the brain as counter electrode), neurons were labeled by electroporation. To label LNs, we used the same method, but the micropipette was directed to the central fiber core in the ordinary glomerular region (OGR) which is densely innervated by neurites of LNs [23]. Physiological experiments were started after a 2–4 h incubation period at room temperature. Imaging was performed with a CCD camera (iXon^EM+^ EMCCD DU-897E; Andor Technology) using 20× (XLUMPFLN 20XW, N.A. 1.0; Olympus), 40× (LUMPLFLN 40XW, N.A. 0.8; Olympus), or 60× (LUMPLFLN 60XW, N.A. 0.9; Olympus) water immersion lenses on a fluorescence microscope (BX51WI; Olympus) with U-MWIBA3 filter set (Olympus). Images were acquired at 10 Hz with 99.8 ms exposure time and an interval between trials of at least 1 min. After imaging, the brains were fixated in 4% paraformaldehyde overnight at 4^°^C, dehydrated through a graded ethanol series, and cleared in methyl salicylate. Labeled neurons were identified morphologically using a confocal laser scanning microscope (LSM-510; Carl Zeiss) with 40×/1.0 oil immersion lens. Confocal images were adjusted for contrast and brightness using Adobe Photoshop (Adobe Systems).

### Loose-patch Recording

The recording of PNs was performed using micropipettes (3–5 MΩ) filled with Alexa Fluor 568 (A-10441; Molecular Probes, 0.1 mM in saline) for visualization. To increase the quality of the seal between micropipettes and PNs, the brain was treated with enzymes (collagenase, 038-10531; Wako, 0.5 mg/ml, dispase, D4693; Sigma, 2 mg/ml, in saline) at room temperature for 5–10 min [24]. Bombykol responsive PNs were identified by applying 10 ng bombykol as a diagnostic stimulus after loose-patch formation. For simultaneous loose-patch recording and calcium imaging, we loaded PNs with calcium indicator as described before, and selected a labeled soma for loose-patch recording. Voltage was amplified (MEZ-8300; Nihon Kohden), low-pass filtered at 5 kHz, and digitized at 10 kHz (USB-6009; National Instruments).

### Stimulation

Synthetic (E,Z)-10,12-hexadecadien-1-ol (bombykol), the principal pheromone component of *B. mori* was diluted with n-hexane (085-00416; Wako) from a stock solution (1 µg/µl n-hexane). A glass cartridge (5 mm inner diameter) was prepared for stimulation by inserting a piece of filter paper (1×2 cm) containing 5 µl odorant solution or n-hexane as control. The odorant was delivered by switching a 3/2 solenoid valve (YDV-3-1/8; Takasago Electric) with constant air flow (1 l/min) from control to pheromone cartridge. Male silkmoths encounter stimulus concentrations of up to a few thousand ng bombykol in the presence of a female (unpublished data from our laboratory), therefore, we used 1–5000 ng as the range of stimulus concentrations.

### Pharmacology

Picrotoxin (PTX, 168-17961; Wako) was prepared as a 250 mM stock in dimethyl sulfoxide and used at a final concentration of 50 µM in silkmoth saline. Neuronal responses were measured after bath application of the drug for 10 min.

### Data Analysis

**Figure 1:**
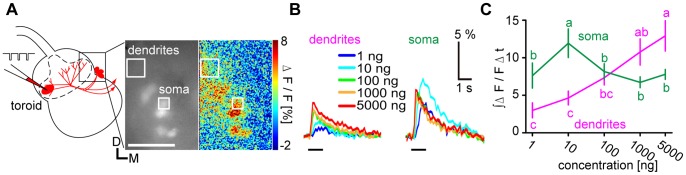
Different concentration-response characteristics in dendrites and somata of antennal lobe (AL) projection neurons (PNs). **A,** Schematic diagram of loading a calcium indicator into PNs with a micropipette by local electroporation (left). The toroid glomerulus processing bombykol is delineated by a dashed line. Fluorescence images of labeled PNs (middle) and the response to 1000 ng bombykol in false colors (right) are shown. Dendritic and somatic regions of interest (ROIs) are indicated by boxes. D: dorsal, M: medial. Scale bar: 50 µm. **B,** Representative time courses of PN responses to bombykol stimuli in the dendrites (left) and a soma (right). Black bars under time courses indicate the stimulus. **C,** Concentration-response characteristics of PN dendrites (magenta) and somata (green). Calcium responses were integrated over 3 s from stimulus onset. (P<0.05 for significant differences indicated by different letters associated with the data groups shown as means±SEM, n = 6 for dendrites and n = 17 for somata, one-way repeated measures ANOVA followed by Tukey-Kramer test).

**Figure 2:**
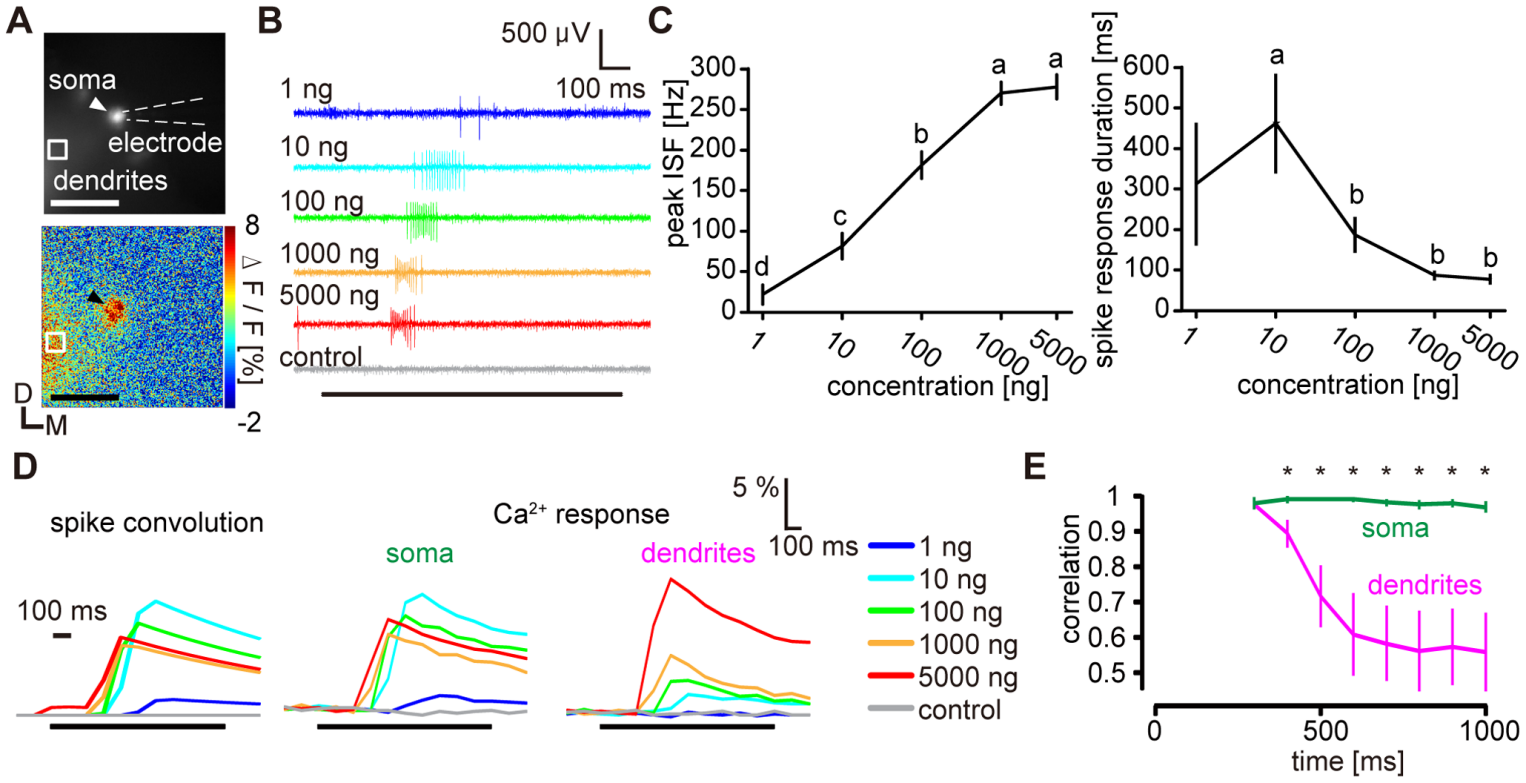
Relation between calcium and spike responses in PNs. **A,** Fluorescence images of PNs labeled with calcium indicator (top) and the response to 1000 ng bombykol in false colors (bottom). Spike responses were recorded from the labeled soma indicated by the arrowhead. The microelectrode for loose-patch recording is delineated by dashed lines and the ROI in the dendritic region is marked. D: dorsal, M: medial. Scale bars: 50 µm. **B,** Representative PN spike responses to bombykol stimuli. The responses were recorded from the soma shown in (A). Black bar under the spike responses indicates stimulus. **C,** Concentration-response characteristics of PN spike responses. The response amplitudes were quantified using peak instantaneous spike frequency (peak ISF, left) and spike response duration (right). (P<0.05 for significant differences indicated by different letters associated with the data groups shown as means±SEM, n = 6, one-way repeated measures ANOVA followed by Tukey-Kramer test). **D,** Representative time courses of convoluted PN spike responses (left) and simultaneously acquired calcium responses in the soma (middle) and the dendrites (right). Same sample as shown in (A) and (B). **E,** Time courses of correlation coefficients between PN spike convolutions and calcium responses in the dendrites (magenta), and between the spike convolutions and calcium responses of the somata (green) (*: P<0.05 between both correlation coefficients at each time point, n = 6, Wilcoxon signed-rank test, data shown as means±SEM). For the first 200 ms following stimulus onset, correlation coefficients are not available because spike responses had a longer latency for all stimulus concentrations.

**Figure 3:**
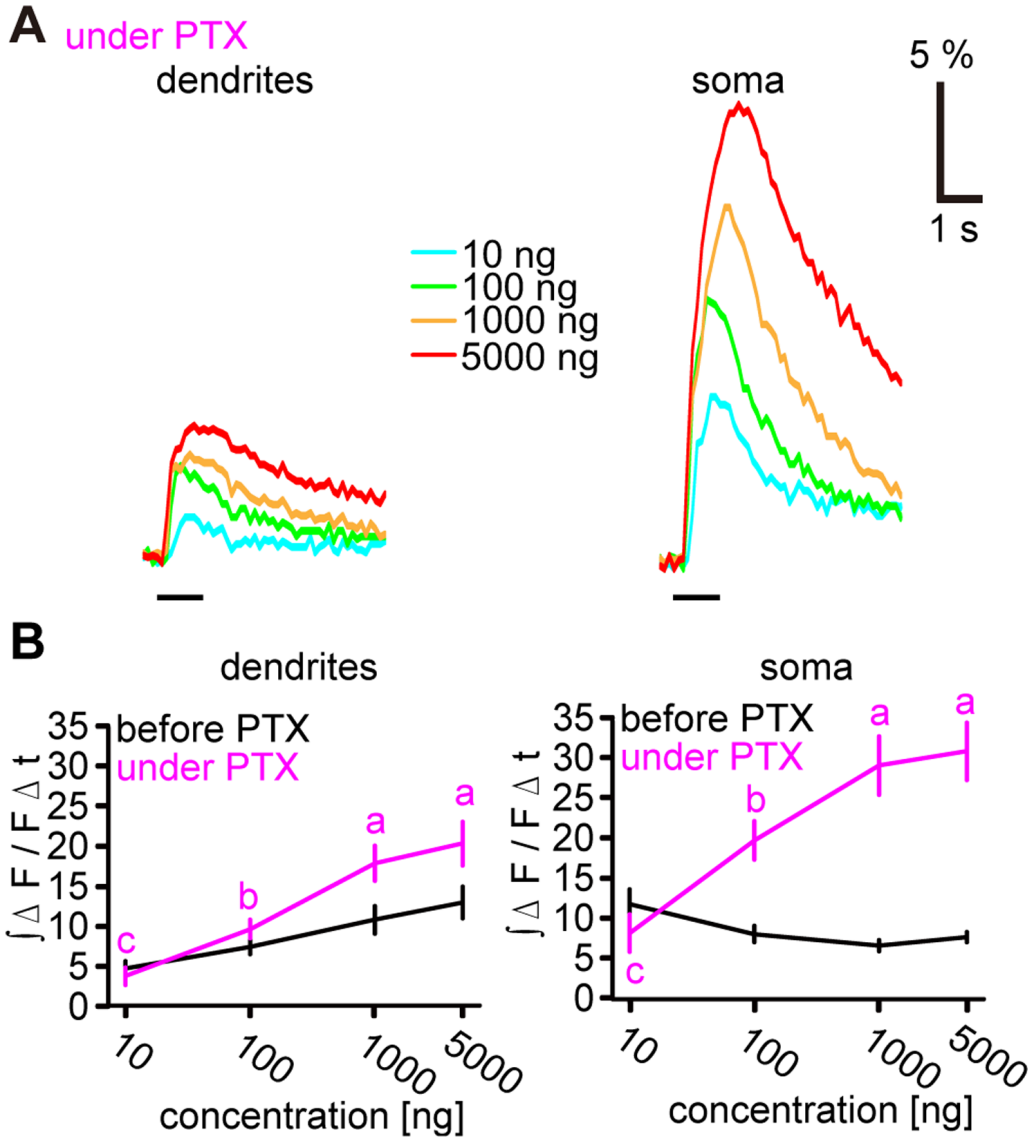
Concentration-response characteristics of PNs under picrotoxin (PTX) treatment. **A,** Representative time courses of PN responses to bombykol stimuli in the dendrites (left) and a soma (right) under PTX treatment. Black bars under time courses indicate stimulus. **B,** Concentration-response characteristics in PN dendrites (left) and somata (right) before (black) and under PTX treatment (magenta). Calcium responses were integrated over 3 s following stimulus onset (P<0.05 for significant differences indicated by different letters associated with the data groups shown as means±SEM, n = 6 at dendrites and n = 17 at somata, one-way repeated measures ANOVA followed by Tukey-Kramer test).

**Figure 4:**
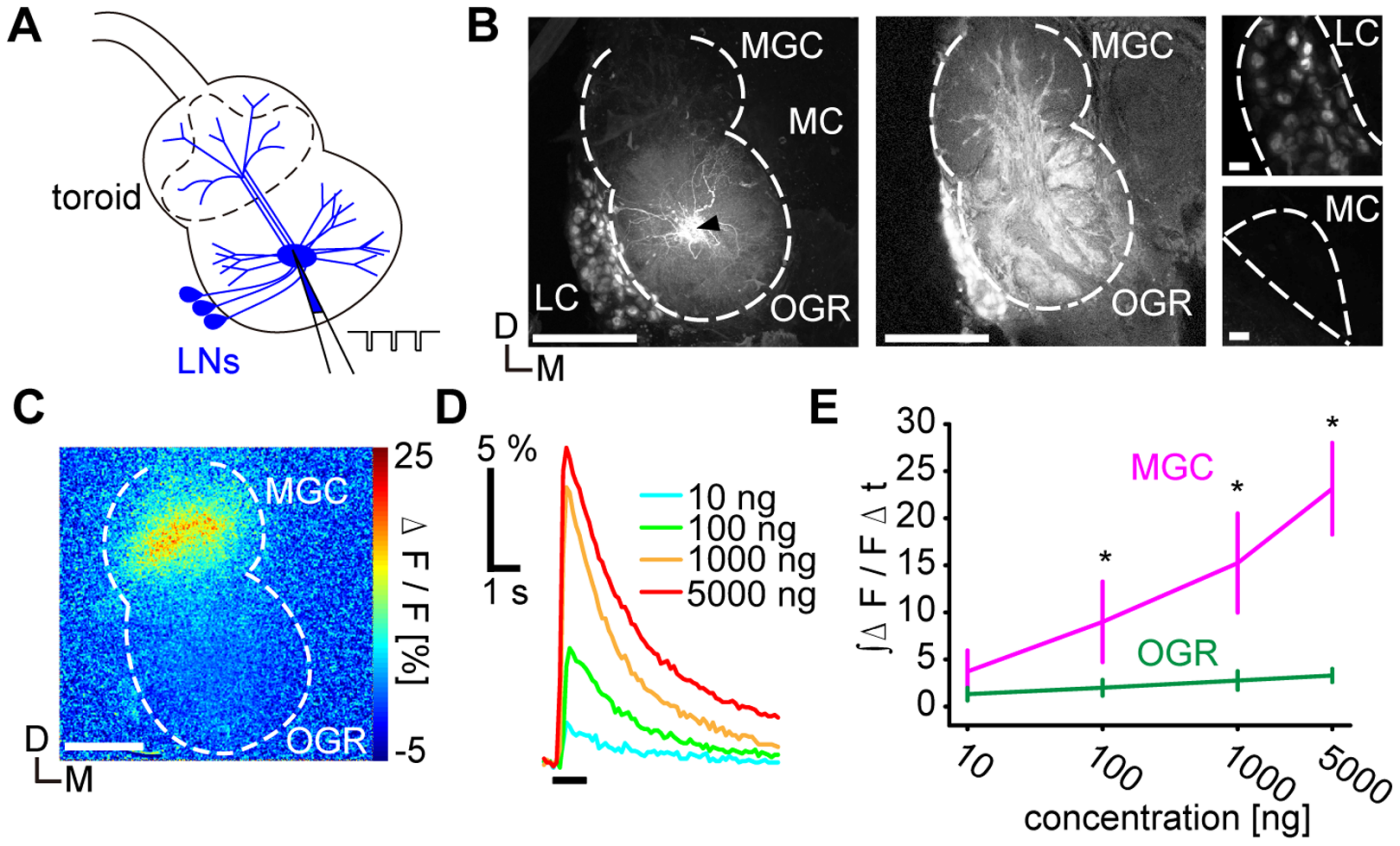
Calcium responses of local interneurons (LNs) to bombykol stimuli. **A,** Schematic diagram of loading the calcium indicator into LNs with a micropipette by local electroporation. The toroid glomerulus is delineated by a dashed line. **B,** Two-dimensional projection of confocal sections (left, extending over 180 µm from the AL surface) and a single confocal optical section (middle) of labeled LNs. Scale bars: 100 µm. The arrowhead in the projection image indicates the injection site. LN branches innervating the MGC are resolved in the optical section. Stack projection images of the medial cell cluster (MC, right, top, extending over 60 µm in depth) and the lateral cell cluster (LC, right, bottom, extending over 60 µm in depth) are enlarged. Brightness and contrast of the images were adjusted at the same level for both cell clusters. The outlines of both cell clusters are delineated by dashed lines. Scale bars: 10 µm. **C,** Representative calcium response of LNs to 5000 ng bombykol in false colors. **D,** Representative time courses of LN calcium responses, same sample as in (C). Black bar under time courses indicates stimulus. **E,** Concentration-response characteristics of LNs in MGC (magenta) and in the OGR (green). Calcium responses were integrated over 3 s following stimulus onset (*: P<0.05, between both regions at given concentration, n = 7, Wilcoxon signed-rank test, means±SEM). D: dorsal, M: medial, MGC: macroglomerular complex, OGR: ordinary glomerular region. The outline of the AL is delineated by dashed lines.

Data were processed using MATLAB (The MathWorks) and Excel (Microsoft). For imaging, per-pixel baseline fluorescence was determined as an average over 10 frames (1 s) before stimulation and relative fluorescence changes (ΔF/F) with respect to baseline fluorescence were calculated. Data were spatially averaged in regions of interests (ROIs, somata: 12×12 µm, neuropil: 20×20 µm in Fig. 1, 13×13 µm in Fig. 2, 20×20 µm in Fig. 3, 39×39 µm (MGC) and 156×156 µm (OGR) in Fig. 4). The exponential decay due to bleaching was removed by subtracting control stimulus trials from the responses. Occasionally, LNs also responded to control stimuli, possibly due to mechanosensory stimulus artifacts [25]. PNs never responded to control stimuli. Temporally integrated neuronal activities (∫ ΔF/F Δt) were calculated by cumulating ΔF/F over 3 s from stimulus onset. A gaussian filter was applied to the images with a five pixel kernel (σ = 1).

Loose-patch recording data from PNs sampled at 10 kHz were high-pass filtered at 100 Hz (1-pole Butterworth) and then smoothed (five-point moving average). Spike response duration was defined as the duration between the time of first and last spikes to an odorant stimulus. The responses to bombykol were always phasic and spontaneous spikes were rarely seen, therefore, we could simply calculate peak instantaneous spike frequencies (ISFs) and spike response durations. Some PNs did not respond reliably to 1–10 ng bombykol. Therefore, these concentrations were excluded from the analysis of response duration.

For comparing simultaneously acquired calcium and spike responses, we estimated calcium fluorescence changes from spike trains by convolution with a kernel described as A×exp(t/−τ) where A is the spike amplitude (peak-to-peak), t is time and τ is the time constant ( = 1.5 s, estimated from the time constant of calcium responses). The estimated values were integrated over 100 ms bins to match the frame rate of calcium imaging. For evaluation, we calculated the correlation between estimated and recorded calcium responses from a data set including 6 stimulus concentrations per experiment separately for 8 bins of 100 ms width after response onset.

Unless otherwise noted, a one-way repeated measures ANOVA followed by the Tukey-Kramer test was used to compare multiple data groups, and the Wilcoxon signed-rank test was used for paired comparisons.

## Results

### Calcium Responses of PNs have Different Concentration-response Characteristics in Dendrites and Somata

To investigate how bombykol concentration affects PN responses in the male silkmoth AL, we specifically loaded PNs innervating the toroid (henceforth just PNs) with the calcium indicator by local electroporation (Fig. 1*A*) [22]. Imaging enabled us to observe the PN responses to bombykol both in the dendrites and the somata (Fig. 1*A*). For convenience, we defined 1–10 ng as “low” bombykol stimulus concentration and 100–5000 ng as “high” concentration. In the dendrites, PN responses increased in a concentration-dependent manner (Fig. 1*B,C*). However, at the somata, the responses decreased at high odorant concentrations (Fig. 1*B,C*). This implies that the dose-response curve of PN calcium responses is modified downstream of the ORN-PN synapses specifically at high odorant concentrations.

### Calcium Responses of PN Somata Reflect Cumulated Spike Information

For the interpretation of calcium responses, the first question is how dendritic and somatic calcium signals are related to spiking activity. To answer this question, we simultaneously performed calcium imaging and loose-patch recording in PNs (Fig. 2*A,B*). The peak ISF of PNs increased in a concentration-dependent manner while the duration of the spike response decreased at high concentrations compared to low concentrations (Fig. 2*B,C*). To compare the spike response patterns with simultaneously acquired calcium-related responses, we transformed the spike trains into estimated calcium fluorescence changes by convolution. Spike amplitudes sometimes changed within spike trains and we compensated the changes based on the assumption that calcium influx at the somata occurs through voltage-gated calcium channels [26]. Representative time courses of spike convolutions and simultaneously measured calcium responses of dendrites and a soma are shown in Fig. 2*D*. Time courses of estimated calcium signals using spike convolution were highly correlated with actual calcium responses of the corresponding somata (Fig. 2*D,E*, r >0.96 on average for every 100 ms bin of the responses) but correlations with calcium responses of dendrites were much lower (Fig. 2*D,E*). This indicates that calcium responses of PN somata rather than of the dendrites reflect spike information in these neurons. The relationship between spike response patterns and calcium responses also implies that the decrease of somatic calcium responses at higher odorant concentrations results from a decrease of the durations of spike responses.

### Inhibition Shapes Concentration-response Characteristics of Somatic PN Calcium Responses

In contrast to somatic responses, calcium responses of PN dendrites showed a monotonic increase with bombykol concentration (Figs. 1, 2). Previous observations in insect brains suggest that calcium responses of dendrites result from calcium entry through nicotinic acetylcholine receptors while calcium influx at the somata is mainly due to voltage-gated calcium channels [26]–[28]. This could explain the differences in calcium responses of PN dendrites and somata in our experiments. Differences between signals originating from excitatory postsynaptic potentials in the dendrites and spike-related signals can also be due to the properties of the spike generator of the PNs themselves, or to inputs from other AL neurons to the PNs downstream of the ORN-PN synapses. After applying picrotoxin (PTX), a γ-aminobutyric acid type A receptor (GABA_A_R) blocker, calcium responses of the PN somata increased at high concentrations (P<0.01, PTX against control at 100, 1000 and 5000 ng, n = 17 somata from 6 animals) and showed a concentration-dependent increase similar to the dendritic responses (Fig. 3*A,B*). Like the somatic calcium responses (Fig. 2), spike response durations were increased following high concentration odorant stimuli under PTX treatment (Fig. S1). Therefore, somatic calcium increase is implied to result chiefly from the lengthening of spike responses under these conditions. These results also show that PN spike responses which are reflected by calcium responses of the somata are controlled by inhibition via GABA_A_Rs downstream of the ORN-PN synapses depending on the odorant stimulus concentration. Some response increase was also observed in the dendrites for high odorant concentrations (Fig. 3*A,B*, P<0.05, PTX against control at 1000 and 5000 ng, n = 6), suggesting that PTX-sensitive inhibitory mechanisms may also act either on the pre- or postsynaptic side of the ORN-PN synapses.

### LN Responses to Bombykol are Spatially Confined and have a High Threshold

GABAergic inhibition is due to LNs in the silkmoth AL [23], [29]. Responses of LNs to bombykol were recorded by calcium imaging [22]. A micropipette filled with the calcium indicator was positioned into the center fiber core of the ordinary glomerular region (OGR) that is densely innervated by neurites of LNs [23], and loaded these neurons with the dye by local electroporation (Fig. 4*A*). In the MGC, the arborizations of labeled neurons were restricted to the cores of the glomeruli, to which LN projections are restricted (Fig. 4*B*) [23]. In addition, labeled somata were localized almost exclusively in the lateral cell cluster (LC, Fig 4*B*), which demonstrates that LNs were labeled with high specificity [21], [29]. Labeled neurites both in the MGC and the OGR therefore belong to LNs. Interestingly, detectable LN responses to bombykol were confined to a subregion of the MGC corresponding to the toroid despite the fact that the LNs also innervate numerous other glomeruli across wide areas of the AL (Fig. 4*C,E*) [29]. Responses of LNs to bombykol were concentration-dependent above a rather high threshold, which coincided with the threshold for response decrease in PNs (Figs. 1*C* *,* 4*D,E*). These results imply that LNs intraglomerularly modulate PN responses in a concentration-dependent manner.

## Discussion

### Relationship between Calcium and Spike Responses

In previous calcium imaging studies investigating PN concentration-response characteristics in insect ALs, calcium responses were mostly observed in dendritic regions [25], [30]–[33]. We demonstrated in a moth pheromone circuit that the calcium changes at the somata, but not in the dendrites reflect spike information (Fig. 2). Furthermore, we showed that spike response duration rather than peak ISF of PNs is affected by intraglomerular inhibition in the AL (Fig. 3 and Fig. S1). The spike generator of PNs is thought to be located where all dendritic branches join to form a single neurite [34] and there is no evidence for a separate dendritic spike generator. The low spike amplitude in MGC recordings from several species of moths supports the idea that the spikes observed in the dendrites are predominantly electronically backpropagated from the downstream spike generator [17]–[19], [21], [30], [35]. Therefore, calcium responses of the dendrites are likely to mainly reflect synaptic potentials rather than spike activity, which is consistent with our results. The increased incidence of retrogradely propagated spikes could be a source of calcium response increase at PN dendrites under PTX treatment. Calcium response increase at PN somata, however, directly reflects the activity of the spike generator under all experimental conditions.

The observation that AL neuron response duration does not increase monotonously with stimulus concentration while peak ISF does was also made in other moth species [17], [20], [35]. Similar mechanisms are likely to be present in other insect species. In the honeybee, calcium responses of PN axon terminals to stimulation with general odorants sometimes decrease with increasing concentration [36] while calcium responses in dendritic regions of PNs generally show a concentration-dependent increase [33]. Although Root et al. demonstrated that calcium responses of PN somata are correlated with the responses of their dendrites in the fruit fly AL when analyzing the specificity of odorant tuning, they also found that spike responses associated with the VM2 glomerulus decreased at high odorant concentrations [37]. Postsynaptic inhibition specific to high odorant concentrations as shown in our present results is therefore likely to be a general feature in odorant processing in the AL.

Similarly to the analogy of lateral inhibition implemented by LNs in the insect AL [8], [11] and by granule cells in the mammalian olfactory bulb [38], [39], an analogy can be drawn between the inhibitory mechanism found in our study and the effect of (postsynaptic) inhibition originating from periglomerular cells acting at intraglomerular level on external tufted and mitral/tufted cells via GABA_A_R in the mammalian olfactory bulb [40].

### Functional Role of Postsynaptic Inhibition

Possible functional roles for postsynaptic inhibition are the maintenance of a concentration-invariant response for preserving odorant identity [8], [41], [42] or the prevention of response saturation [8] especially at higher concentrations. However, our results show that inhibition shortens PN spike response duration rather than affecting peak ISF. Therefore, an alternative and more plausible function of inhibition is an involvement in the control of temporal response patterns of PNs, that become important when encountering pulse trains of odorant stimuli, as present under natural conditions. The involvement of inhibition in shortening response durations has previously been demonstrated in the sphinx moth, *Manduca sexta* [43]–[46]. We have now shown that inhibition involved in response shortening acts downstream of the ORN-PN synapses and in a concentration-dependent manner. Concentration-dependent increase of PN spike response duration under PTX treatment might partially arise from slow offset kinetics of odorant stimulation, in particular at high concentrations (e.g., hysteresis of the stimulator system, odorant vapor pressure). Even if such an effect were present, it would not affect our major finding concerning the function of inhibition at high concentrations because inhibition is activated only at high concentrations and shuts off PN responses well before nominal stimulus offset (Fig. 2). Inhibition acting specifically at higher stimulant concentrations would maintain short response durations over a wide concentration range while preserving odorant detectability at lower concentrations.

### Localization of LN Activity

Bombykol stimuli induced strong responses in LNs localized in a subregion of the MGC although LNs innervate a large number of other glomeruli as well [29]. In our paradigm, multiple LNs were labeled and we could not discriminate individual LN responses. However, LNs exclusively innervating the MGC are unlikely to exist [29]. This implies that the neuronal responses are confined to subcellular compartments of multiglomerular LNs that innervate the MGC and supply strictly intraglomerular inhibition rather than being a substrate for interglomerular interactions for processing odorant mixtures.

In ordinary glomeruli, LN branches are found throughout the entire volume of each glomerulus whereas LN arborizations were absent from the cortex of silkmoth MGC glomeruli (Fig. *4B*) [23], that is innervated by ORNs [47]. As in the case of LN1 cells in the fruit fly AL, that only innervate the cores of glomeruli [48], LNs are likely to predominantly target PNs rather than ORNs in the MGC. In our study, dendritic responses of PNs were strictly concentration-dependent while somatic and spike responses were suppressed at higher stimulus concentrations. It is therefore likely that LNs synapse onto PNs in the core of the glomerulus where the spike generator of PNs is thought to be located [34]. Intraglomerular inhibition would therefore act at the level of the spike generator rather than on excitatory postsynaptic potentials.

In summary, we found that locally confined postsynaptic inhibition in the AL shortens PN spike response duration specifically at high stimulus concentrations controlling the temporal representation of olfactory signals that is important for odorant source localization under natural conditions [49].

## Supporting Information

Figure S1 **Spike response durations of PNs under PTX treatment.** **A,** Representative PN spike responses to bombykol stimuli under PTX treatment. Black bar under the spike responses indicates stimulus. **B,** Concentration-response characteristics of PN spike responses quantified using spike response duration (P<0.05 for significant differences indicated by different letters associated with the data groups shown as means±SEM, n = 7, one-way repeated measures ANOVA followed by Tukey-Kramer test).
(TIF)
