# Supplementary figures and images for: Postsynaptic Odorant Concentration Dependent Inhibition Controls Temporal Properties of Spike Responses of Projection Neurons in the Moth Antennal Lobe
Source: PLoS One. 2014 Feb 19;9(2):e89132. doi: 10.1371/journal.pone.0089132 (PMC3929629; doi:10.1371/journal.pone.0089132)

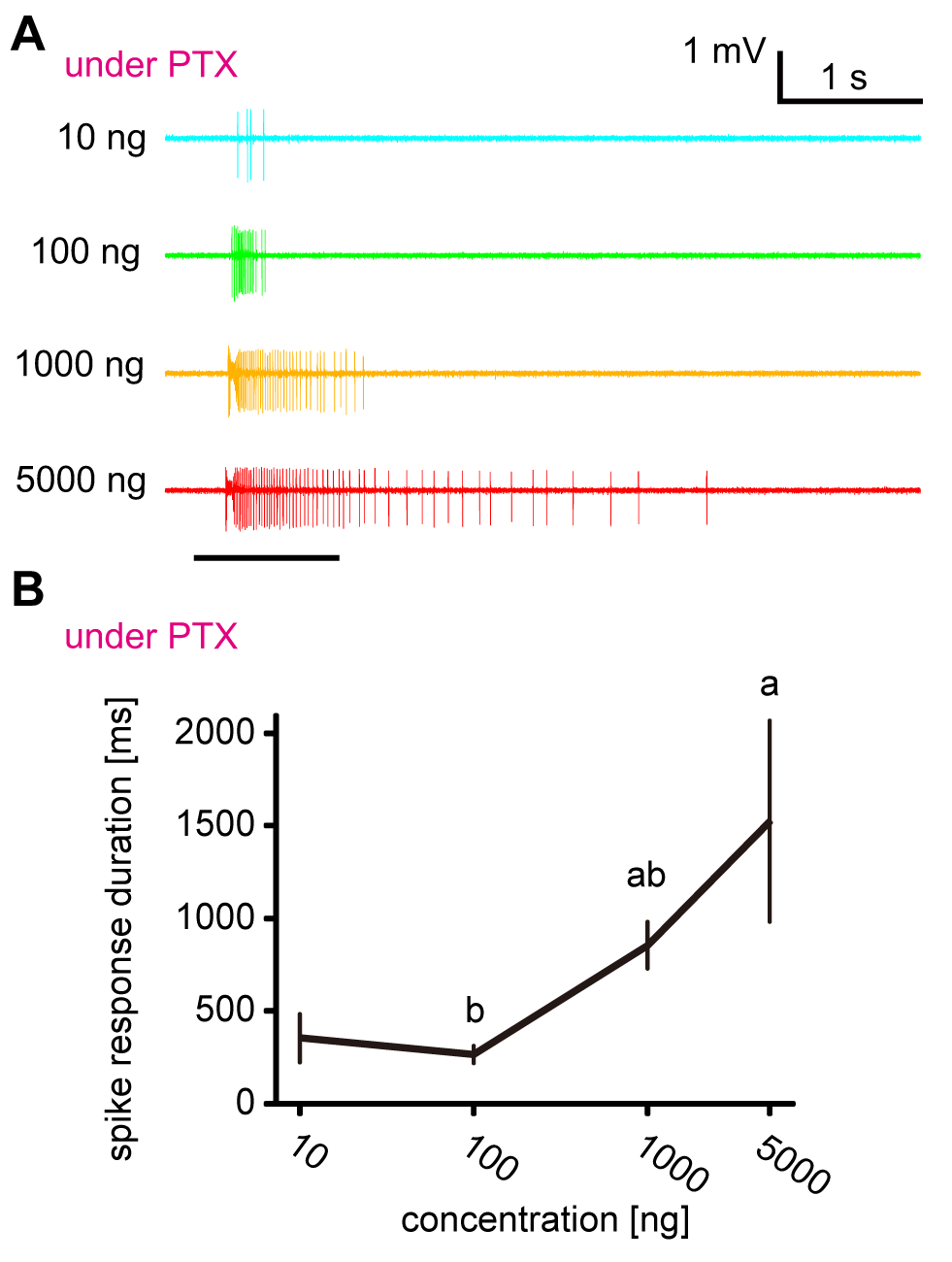

Supplement: Figure S1 — Spike response durations of PNs under PTX treatment. A, Representative PN spike responses to bombykol stimuli under PTX treatment. Black bar under the spike responses indicates stimulus. B, Concentration-response characteristics of PN spike responses quantified using spike response duration (P<0.05 for significant differences indicated by different letters associated with the data groups shown as means±SEM, n = 7, one-way repeated measures ANOVA followed by Tukey-Kramer test). (TIF) [file pone.0089132.s001.tif]
